# Balloon‐Expandable Versus Self‐Expanding Valves in Low‐Flow, Low‐Gradient Aortic Stenosis: Does Valve Type Matter?

**DOI:** 10.1002/ccd.70529

**Published:** 2026-02-23

**Authors:** Nav Warraich, James A. Brown, Abbad Sultan, Dustin Kliner, Derek Serna‐Gallegos, Catalin Toma, Amber Makani, Irsa Hasan, Takuya Ogami, Jianhui Zhu, Ibrahim Sultan

**Affiliations:** ^1^ Department of Cardiothoracic Surgery, Division of Cardiac Surgery University of Pittsburgh Pittsburgh Pennsylvania USA; ^2^ Heart and Vascular Institute University of Pittsburgh Medical Center Pittsburgh Pennsylvania USA; ^3^ Department of Medicine, Division of Cardiology University of Pittsburgh Pittsburgh Pennsylvania USA

**Keywords:** aortic valve disease, balloon‐expandable, low‐flow low‐gradient aortic stenosis, self‐expanding, TAVR, transcatheter aortic valve replacement

## Abstract

**Background:**

The optimal choice between balloon‐expandable (BE) and self‐expanding (SE) transcatheter heart valves (THVs) in patients with low‐flow low‐gradient aortic stenosis (LFLG AS) undergoing transcatheter aortic valve replacement (TAVR) remains unclear.

**Aims:**

This study aims to compare outcomes of BE THVs and SE THVs in patients with LFLG AS.

**Methods:**

This was a retrospective, single‐institution cohort study of TAVR procedures between 2012 and 2024. Patients with LFLG AS (mean gradient < 40 mmHg and ejection fraction < 50% or stroke volume index < 35 mL/m^2^) were stratified by respective valve type. Patients with prior aortic valve replacement were excluded. Postoperative outcomes were compared between groups. Kaplan−Meier estimates and a Cox regression model were generated for survival. Cumulative incidence curves and a cause‐specific cox regression model were generated for heart failure (HF) readmission.

**Results:**

A total of 732 patients identified underwent TAVR for LFLG AS. Of these, 478 (65.3%) received a BE THV and 254 (34.7%) received a SE THV. STS PROM was similar between groups. There was no difference in 30‐day mortality between groups. SE THVs had a lower mean gradient but increased rates of paravalvular leak at 30 days post‐TAVR. Kaplan−Meier estimates were comparable between groups and there was no significant difference after multivariable analysis (HR 1.15, 95% CI 0.90−1.46, *p* = 0.27). Cumulative incidence estimates of HF readmission were similar between groups, however, BE THV was associated with reduced hazard of HF readmission on multivariable analysis (HR 0.77, 95% CI 0.59−0.99, *p* = 0.04).

**Conclusions:**

In patients with LFLG AS undergoing TAVR, BE and SE THVs have comparable midterm survival. BE THVs have a reduced risk of HF readmission on multivariable analysis.

## 1 Introduction

A subset of patients with severe aortic stenosis (valve area < 1.0 cm^2^) present with low transvalvular pressure gradients (< 40 mmHg) and have low‐flow, low‐gradient aortic stenosis (LFLG AS) [1]. LFLG AS can be further stratified into “classical” with reduced ejection fraction (< 50%) or “paradoxical” with low‐flow (SVI < 35 mL/m^2^) despite preserved ejection fraction (≥ 50%). These types of LFLG AS reflect systolic and diastolic ventricular dysfunction, respectively, and are associated with poorer prognosis compared to high‐gradient aortic stenosis [2, 3, 4]. Aortic valve replacement (AVR) has been shown to provide significant survival benefit in patients with LFLG AS [5, 6, 7, 8, 9, 10], however, there is limited data comparing specific interventions. Thus, ACC/AHA guidelines [11] recommend AVR in LFLG AS; however, no preference is given between transcatheter and surgical approaches, nor is a specific valve type endorsed.

Although a significant portion of patients with LFLG AS undergo transcatheter aortic valve replacement (TAVR), it is unclear if there is an optimal valve type. In the general population of patients undergoing TAVR, self‐expanding transcatheter heart valves (SE THVs) optimize hemodynamics with reduced post‐TAVR gradients [12, 13] but have increased rates of paravalvular leak (PVL) [14, 15, 16] compared to balloon‐expandable transcatheter heart valves (BE THVs). Additionally, SE THVs have been associated with increased rates of permanent pacemaker implantation [14, 15, 17]. Despite these differences, randomized and meta‐level data have found comparable survival between BE and SE THVs [15, 16, 18]. Thus, guidelines suggest a patient‐specific approach to selection of valve type taking into consideration anatomical and clinical factors [11]. In patients with LFLG AS, however, there are few studies comparing valve types with limited sample size [19, 20]. Due to differences in baseline ventricular function and hemodynamics, the effect of valve type in this subpopulation warrants investigation.

Therefore, this study aims to compare outcomes of BE THVs and SE THVs in patients with LFLG AS.

## 2 Patients and Methods

### 2.1 Population and Study Design

This was a retrospective, single institution cohort study, utilizing a prospectively maintained institutional database at the University of Pittsburgh Medical Center (UPMC) of patients who underwent TAVR from 2012 to 2024. Patients who had a prior AVR were excluded. Patients with LFLG AS were divided into two groups by those receiving a SE THV and those receiving a BE THV. Data was extracted from preoperative resting transthoracic echocardiography. LFLG AS was defined as an aortic valve area ≤ 1.0 cm^2^ with an aortic valve mean gradient < 40 mmHg and a stroke volume index < 35 ml/m^2^. The primary outcomes of interest were survival and heart‐failure readmission. Secondary outcomes included postoperative and echocardiographic outcomes. Post‐TAVR gradients were measured using transthoracic echocardiography. Follow‐up data was obtained from the UPMC clinical warehouse that contains all longitudinal survival data for patients undergoing cardiac operations at UPMC. This study was approved by the Institutional Review Board of the University of Pittsburgh (STUDY1812043; April 17, 2019), with written consent being waived.

### 2.2 Statistical Methods and Analysis

Primary stratification was by valve type. Continuous variables were presented as mean ± standard deviation if distributed normally. Non‐normally distributed continuous variables were presented as median and interquartile range. Categorical data was presented as frequency and percentage. Variables were compared between groups. Student's *t*‐test was used for normally distributed continuous variables, and the nonparametric Mann−Whitney *U* test was used for non‐normally distributed continuous variables. Chi‐squared or Fisher's exact test was used to compare categorical variables, as appropriate.

Baseline characteristics of groups were compared. Postoperative outcomes and echocardiographic outcomes at 30 days were determined. Survival estimates were generated using Kaplan−Meier methods and compared by the log‐rank test. Cumulative incidence curves were generated for heart failure readmission and compared by Gray's K‐sample test. Kaplan−Meier and cumulative incidence curves were also generated comparing valve type for the classical and paradoxical subtypes of LFLG AS. A multivariable Cox proportional‐hazards regression model for the overall cohort was generated to assess variables associated with overall survival. A cause‐specific Cox proportional‐hazards regression model was generated to identify variables associated with heart‐failure readmission. Models were built using backwards stepwise selection with a *p* value threshold of inclusion ≤ 0.20. All variables in Table 1 were considered for model building except STS‐PROM as it involves baseline comorbidities already included. All tests were completed as two‐sided with an *⍺* level of 0.05 to determine statistical significance.

**Table 1:** Baseline variables.

Variable	BEV (*n* = 478)	SEV (*n* = 254)	*p* value
Age	80.3 ± 7.69	82.2 ± 7.21	0.001
Female	79 (16.53%)	63 (24.80%)	0.01
White race	422 (88.28%)	223 (87.80%)	0.85
Body mass index	29.3 ± 6.60	28.2 ± 6.54	0.03
STS PROM	6.94 ± 5.67	6.67 ± 4.95	0.52
Atrial fibrillation	257 (53.77%)	143 (56.30%)	0.51
Hypertension	433 (90.59%)	227 (89.37%)	0.60
Diabetes	234 (48.95%)	107 (42.13%)	0.08
Chronic dialysis use	19 (3.97%)	7 (2.76%)	0.40
NYHA functional class			0.01
I	8 (1.67%)	4 (1.57%)	
II	111 (23.22%)	82 (32.28%)	
III	268 (56.07%)	141 (55.51%)	
IV	91 (19.04%)	27 (10.63%)	
Peripheral vascular disease	117 (24.48%)	100 (39.37%)	< 0.001
Cerebrovascular disease	58 (12.13%)	31 (12.20%)	0.99
Chronic lung disease	134 (28.03%)	86 (33.86%)	0.10
Prior MI, CABG, or PCI	318 (66.53%)	165 (64.96%)	0.67
paradoxical LFLG	146 (30.54%)	98 (38.58%)	0.03
Aortic valve annular diameter (mm)	25.2 ± 3.81	24.6 ± 2.26	0.05
Implanted valve size (mm)	26.6 ± 2.26	29.4 ± 2.72	< 0.001
Transfemoral vascular access	450 (94.14%)	196 (77.17%)	< 0.001
Baseline EF	41.0 ± 13.9	42.9 ± 14.7	0.09
Baseline AV mean gradient	32.1 ± 7.56	31.8 ± 7.59	0.52
Mitral regurgitation ≥ moderate	119 (24.90%)	49 (19.29%)	0.09
Tricuspid regurgitation ≥ moderate	166 (34.73%)	77 (30.31%)	0.23
Bicuspid aortic valve	16 (3.35%)	2 (0.79%)	0.04

Abbreviations: AV, aortic valve; BEV, balloon‐expandable valve; CABG, coronary artery bypass grafting; EF, ejection fraction; MI, myocardial infarction; NYHA, New York Heart Association; PCI, percutaneous coronary intervention; SEV, self‐expanding valve; STS PROM, Society of Thoracic Surgeons Predicted Risk of Mortality.

## 3 Results

### 3.1 Baseline Variables

A total of 4913 patients without prior AVR underwent TAVR during the study's time frame. Of these 4913 patients, 732 (14.9%) had LFLG AS. Of the 732 patients, which comprised the study's cohort, 478 (65.3%) received a BE THV and 254 (34.7%) received a SE THV. Table 1 presents demographic and clinical data across each group. The BE THV group was younger, with fewer female patients, and had a larger BMI than the SE THV group. The BE THV group also had a greater incidence of bicuspid aortic valve and transfemoral vascular access. The SE THV group had a greater incidence of peripheral vascular disease, paradoxical LFLG AS, and a larger mean implanted valve size.

### 3.2 Post‐TAVR and Echocardiographic Outcomes

Table 2 compares postoperative and echocardiographic outcomes between groups. 30‐day mortality, stroke, atrial fibrillation, permanent pacemaker placement, and major vascular complications did not differ between the BE THV and SE THV groups. At 30 days, the BE THV group had an increased mean gradient but a lower rate of PVL compared to the SE THV group. There was no difference in ejection fraction between groups.

**Table 2:** Postoperative and echocardiographic outcomes.

Variable	BEV (*n* = 478)	SEV (*n* = 254)	*p* value
30‐day mortality	10 (2.09%)	7 (2.76%)	0.57
Stroke	7 (1.46%)	7 (2.76%)	0.23
Atrial fibrillation	6 (1.26%)	3 (1.18%)	1.00
Pacemaker	36 (7.53%)	27 (10.63%)	0.16
Major vascular complication	3 (0.63%)	0 (0.00%)	0.56
	***n*** = **459**	***n*** = **246**	
30‐day EF	46.2 ± 14.3	47.3 ± 13.7	0.35
30‐day AV mean gradient	10.0 ± 4.16	6.73 ± 3.49	< 0.001
30‐day paravalvular leak			0.001
Mild	87 (18.95%)	70 (28.46%)	
Moderate	7 (1.53%)	10 (4.07%)	

Abbreviations: AV, aortic valve; BEV, balloon‐expandable valve; EF, ejection fraction; SEV, self‐expanding valve.

### 3.3 Survival and Heart Failure Readmission

**Figure 1:**
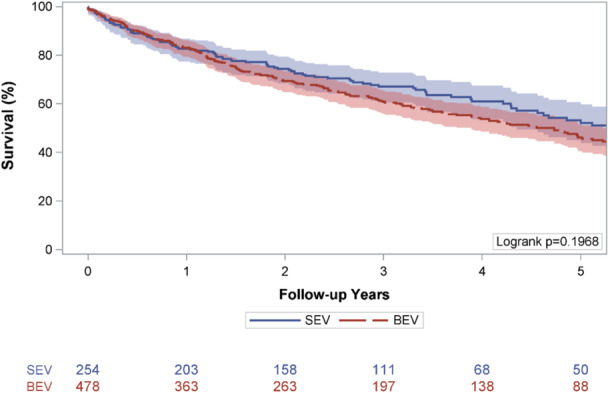
Kaplan−Meier survival estimates. [Color figure can be viewed at wileyonlinelibrary.com]

The median [IQR] follow‐up was 2.38 [1.03−4.42] years in the BE THV group and 2.68 [1.29−4.23] years in the SE THV group (*p* = 0.25). Unadjusted Kaplan−Meier survival estimates were comparable between groups (log‐rank, *p* = 0.20, Figure 1). One‐year survival was 83.1% (79.5−86.1) for the BE THV group, and 82.6% (77.8−87.1) for the SE THV group. Five‐year survival was 46.1% (40.6−51.8) for the BE THV group, and 52.1% (44.1−60.0) for the SE THV group. On multivariable Cox regression, BE THV was not associated with a significant difference in hazard of death compared with SE THV (HR 1.15, 95% CI 0.90−1.46, *p* = 0.27, Table 3). Chronic dialysis use, mitral regurgitation ≥ moderate, tricuspid regurgitation ≥ moderate, diabetes, and paradoxical LFLG AS were associated with an increased hazard of death (*p* < 0.05).

**Table 3:** Multivariable Cox proportional‐hazards model for survival.

Variable	HR	95% CI		*p* value
BEV (ref: SEV)	1.15	0.90	1.46	0.27
Chronic dialysis use	2.88	1.85	4.47	< 0.001
Mitral regurgitation ≥ moderate	1.42	1.11	1.81	0.01
Tricuspid regurgitation ≥ moderate	1.30	1.03	1.65	0.03
Chronic lung disease	1.22	0.96	1.55	0.10
Age	1.02	1.00	1.03	0.05
Diabetes	1.29	1.03	1.61	0.03
Peripheral vascular disease	1.25	0.99	1.57	0.06
Paradoxical versus classical	0.74	0.58	0.95	0.02

Abbreviations: BEV, balloon‐expandable valve; SEV, self‐expanding valve.

**Figure 2:**
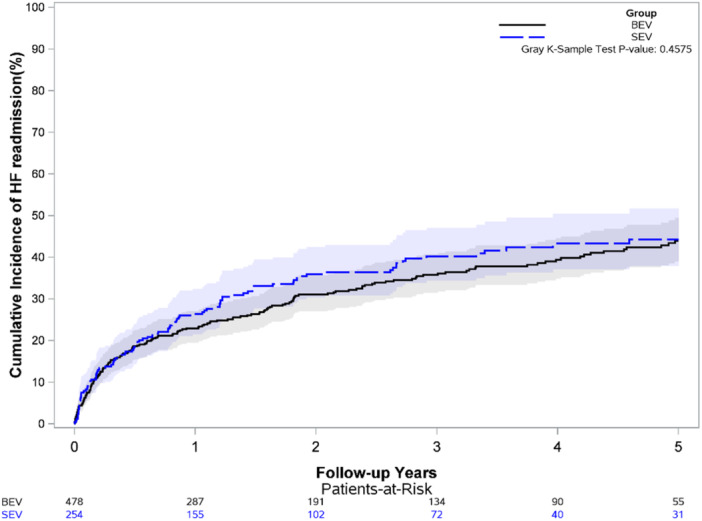
Cumulative incidence of heart failure readmissions. [Color figure can be viewed at wileyonlinelibrary.com]

Cumulative incidence of heart failure readmissions was similar between groups (*p *= 0.46, Figure 2). After multivariable adjustment, however, BE THV was associated with reduced hazard of heart failure readmission compared to SE THV (HR 0.77, 95% CI 0.59−0.99, *p*= 0.04, Table 4). Age, mitral regurgitation ≥ moderate, chronic dialysis use, and atrial fibrillation were also associated with an increased hazard of heart failure readmission (*p* < 0.05).

**Table 4:** Cause‐specific Cox proportional‐hazards regression model for heart failure readmission.

Variable	HR	95% CI		*p* value
BEV (ref: SEV)	0.77	0.59	0.99	0.04
Age	0.98	0.97	1.00	0.04
Mitral regurgitation ≥ moderate	1.64	1.27	2.13	< 0.001
Chronic dialysis use	1.91	1.11	3.28	0.02
Atrial fibrillation	1.35	1.06	1.71	0.02
Transfemoral vascular access	1.33	0.90	1.97	0.16
NYHA functional class (ref: I)				
II	2.83	0.56	14.43	0.21
III	3.55	0.70	17.89	0.13
IV	4.97	0.97	25.36	0.05

Abbreviations: BEV, balloon‐expandable valve; NYHA, New York Heart Association; SEV, self‐expanding valve.

**Figure 3:**
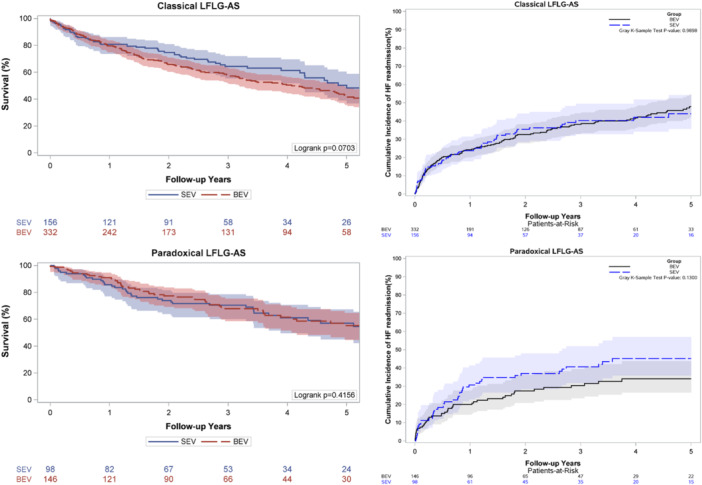
Survival and heart failure readmission stratified by classical and paradoxical LFLG AS subtypes. [Color figure can be viewed at wileyonlinelibrary.com]

In analysis restricted to patients with classical LFLG AS only, those who received BE THVs and SE THVs had no significant difference in mortality or heart failure readmission. This was consistent in the paradoxical LFLG AS subgroup as well which also had no significant difference in mortality or heart failure readmission between valve types (Figure 3).

## 4 Comment

There were several notable findings in this study of patients with LFLG AS undergoing TAVR. First, LFLG AS was present in 14.9% of the total TAVR patients, excluding those with prior AVR. Second, there was no difference in short‐term outcomes, including 30‐day mortality, stroke, and permanent pacemaker placement, between patients receiving BE THVs and those receiving SE THVs. Third, BE THVs had lower rates of PVL but higher residual gradients than SE THVs at 30 days post‐TAVR. Fourth, BE THVs and SE THVs offered comparable midterm survival which was consistent with multivariable analysis. Lastly, there was no significant difference in cumulative incidence of heart failure readmission; however, BE THVs had a reduced risk of heart failure readmission on multivariable analysis.

The incidence of LFLG AS in patients undergoing TAVR has been reported between 13% and 37% in recent studies [21, 22, 23, 24, 25, 26]. Our study reports an incidence of 14.9%. The varying incidence across studies may be attributable to limited sample sizes, patient demographic differences, and inconsistencies in echocardiographic assessment. Regardless, patients with LFLG AS represent a clinically significant subset of patients undergoing TAVR. Despite this, there is limited data comparing BE and SE THVs in this population. To date, only two studies by Covolo et al. [19] and Ishibashi et al. [20] have compared valve type in LFLG AS patients with sample sizes of 146 and 163, respectively. Consistent with findings in these studies and those not restricted to patients with LFLG AS [15, 16, 18], we did not observe a significant difference in 30‐day mortality or stroke between valve types. Previous studies have shown increased permanent pacemaker placement in patients receiving SE THVs [14, 15, 17]. In our study of patients with LFLG AS, pacemaker placement post‐TAVR was observed in 10.6% of SE THV patients and 7.53% of BE THV patients which was not a statistically significant difference. It is important to note, however, that we cannot rule out the association of SE THV with pacemaker placement as there may be inadequate power to detect a difference. Moreover, our findings may be influenced by baseline factors which we were not able to account for such as pre‐existing conduction defects.

Apart from comparable ejection fraction at 30 days, there were distinct echocardiographic findings between the valve types. Post‐TAVR PVL of moderate or greater severity has been consistently linked to reduced survival following TAVR [27, 28], and there is evidence that even mild PVL can reduce survival in the long‐term [28, 29, 30]. Similar to studies examining the general TAVR population [14, 15, 16, 18], increased rates of PVL were observed in patients who received SE THVs for LFLG AS at 30 days post‐TAVR. Lower residual gradients following TAVR with SE THVs compared to BE THVs have been consistently reported in prior studies [12, 13, 14, 15, 16]. Likewise, in our population of LFLG AS patients, lower mean gradients were observed in SE THVs at 30 days. With no clear survival benefit shown in recent studies comparing patients by post‐TAVR gradient [31, 32], the clinical significance of the difference in gradients between valve types in this study is unclear.

Although some observational studies have shown a survival benefit in patients with SE THVs [14, 17], data from randomized trials and a recent comprehensive meta‐analysis have reported no significant difference in survival between valve types [15, 16, 18]. In our LFLG AS population, BE and SE THVs offered comparable survival, and there was no significant difference after multivariable adjustment or stratification by classical versus paradoxical subtypes. In our multivariable model, paradoxical LFLG AS was associated with improved survival compared to classical LFLG AS. This finding is supported by prior studies which have shown that classical LFLG carries the poorest prognosis among AS subtypes [2, 24]. Moreover, baseline mitral regurgitation was associated with a significant reduction in survival and an increase in heart failure readmission. Since mitral regurgitation does not consistently improve after TAVR and has a poor prognosis [33], concomitant mitral intervention may be considered in patients with LFLG AS and ≥ moderate mitral regurgitation.

Interestingly, despite the absence of a survival benefit, BE THVs were associated with reduced heart failure readmission rates compared with SE THVs. This finding suggests that the lower rate of PVL in BE THVs may outweigh the higher transvalvular pressure gradients, compared to SE THVs. This might be particularly true in patients with LFLG AS who may be less able to tolerate the volume overload from PVL due to their pre‐existing left ventricular dysfunction. Multiple observational studies have reported poor outcomes of patients with LFLG AS who develop PVL post‐TAVR [34, 35]. Specifically, a study of the TOPAS‐TAVI registry including over 200 LFLG AS patients found that there was an increased risk of rehospitalization for heart failure in these patients [35]. However, because longitudinal echocardiographic data assessing cardiac function and PVL severity are unavailable, this interpretation remains speculative. In our study, the reduced risk of heart‐failure rehospitalization in the BE THV group did not translate to a decrease in mortality. This may be due to an insufficient time frame or methodological limitations of the study. Ultimately, studies with larger sample sizes, longer follow‐up, and more robust echocardiographic data are needed to investigate BE and SE THVs in patients with LFLG AS.

### 4.1 Limitations

There are several notable limitations to this study. As a retrospective observational study, it is subject to confounding. Additionally, this study included patients at a single high‐volume institution which limits generalizability. A small sample size may have limited power to detect differences in outcomes. Echocardiographic assessment was conducted by cardiologists at different locations, rather than standardized laboratory analysis. 30‐day post‐TAVR mean gradients measured by echocardiography may be subject to measurement bias given differences with invasive gradients. Moreover, missing echocardiographic data may have affected our findings. Advanced assessment using dobutamine stress echocardiography or CT calcium scoring were not performed. Also, we did not have data on cardiovascular death, which may provide a more accurate assessment of valve‐specific effects, given the prevalence of comorbidities in the LFLG AS population. Lastly, loss to follow‐up may introduce bias.

## 5 Conclusion

BE and SE THVs have comparable midterm survival in patients undergoing TAVR for LFLG AS. BE THVs have reduced rates of PVL but increased residual gradients compared to SE THVs post‐TAVR. BE THVs have reduced risk of heart failure readmission on multivariable analysis. Prospective studies with longer follow‐up are needed in this population.

## Funding

The authors received no specific funding for this work.

## Ethics Statement

STUDY18120143 approved, and consent was waived on April 17, 2019.

## Conflicts of Interest

The authors declare no conflicts of interest.

## Data Availability

Data is available from the corresponding author upon reasonable request.
